# Supplementary material for: The Simple One-step stool processing method for detection of Pulmonary tuberculosis: A study protocol to assess the robustness, stool storage conditions and sampling strategy for global implementation and scale-up
Source: PLoS One. 2022 Oct 4;17(10):e0264103. doi: 10.1371/journal.pone.0264103 (PMC9531811; doi:10.1371/journal.pone.0264103)
Supplement: S1 Appendix — (PDF) [file pone.0264103.s001.pdf]

<u>Appendix 1: Informed consent form for PODTEC study participant's parents/guardian) (English version)</u>

Project Title: Diagnostic optimization of stool to diagnose TB in children in Ethiopia

Introduction

Good morning/afternoon/evening.

My name is ____________________ and I am the site coordinator for a study conducted by EPHI and KNCV Tuberculosis Foundation to optimize and standardize the stool processing to diagnose TB in children. The study is supported by KNCV Tuberculosis Foundation in the Netherlands. The project is being conducted at selected facilities in Addis Ababa and the facility you are attending is among the ones selected. The main purpose of this project is to optimize and standardize the stool processing to diagnose TB. If this works diagnosing TB in children becomes much easier and there is no need for invasive methods to obtain sputum. Sputum is normally used to test for TB but for small children or very sick children it is difficult to provide a sputum sample. Children swallow the sputum and therefore currently using a tube the sputum sample is taken through the nose form the stomach, this is not a pleasant procedure for the children.

This project will provide vital information to help in the generation of standard operating procedures (SOPs) for this method for future scale-up in Ethiopia and in other countries. You are asked to participate because your child was identified as having TB by the clinician in this facility.

Study Procedure: We would like to ask you if you are willing for your child to take part in the study. You will be given stool containers to collect the stool. You will submit three days stool samples of your child. We would also like to record some information from the child to help us conduct the analysis, like age, sex, medical condition of the child including the test results obtained from all tests for TB requested by the clinician and the stool results and information on the decision by the clinician on whether or not your child has TB and based on what that decision was taken. We will take the stool sample to the EPHI laboratory to investigate using a machine called GeneXpert whether we can find TB in the stool of the child. Your child is not the only one in this facility who has been asked to participate, all children who are confirmed as TB patients were asked.

Risk and Benefit: There will not be any risk for you and your child if you choose to be part of the project. There may not be direct benefit to you or your child as a participant. However, your participation can help to make diagnosing of TB for children in Ethiopia and other countries simpler

in the future. If you choose not to participate in the study, your child will still receive the standard care and treatment as per the national guidelines. Every person who is part of this study will receive two days transportation allowance will be paid for child and the patients/guardians.

Compensation: Transport money and a compensation will be provided to support the caregiver to make these visits. This will be 150 Ethiopian Birr per day for two days for the extra days that the parents/guardian would travel back and forth to bring/collect stool specimen.

Confidentiality: All the information you give will be kept strictly confidential. We will keep the records in a safe place and only investigator/s will be allowed to look at them. Yours and your child personal identifying information will not be used and shared outside of the study team. We will not use your/your child's name and details for the analysis of the data. Any publications that comes out of the study will not mention any details that will identify you/your child. The study results will facilitate inclusion of simplified child TB diagnostic approaches in the local treatment policy.

Voluntary Participation: You/your child is here for TB treatment based on initial tests, but we want to take more samples to do further studies to improve the method TB detection for other children. Participation in this study is voluntary, you are free to choose for your child to be part of the study and you can decline to participate or remove yourself/your child from the study at any time. You may refuse to answer any of the questions and you may halt your participation at any time. If you decide not to participate in this assessment, or drop out at a later time, your decision will not affect your child's treatment at the facility in any way. If you agree to participate, we would like to ask you to let us take a stool sample from your child on three different days. This sample will be used to perform additional test in the laboratory to find the best method to use stool to detect tuberculosis in children. The results from this study will not have direct effect on the treatment course for your child.

If you have any questions, related to the study please contact the principal investigator of this research Mr. Bazezew Yenew or co-investigator of this research Endale Mengesha and if you have any ethical complaint and issues about by our right, contact Ethic Review Office of Ethiopian Public Health Institute at the following address.

For any questions related to the study or you experienced any adverse event before/during/after participation in the study

Bazezew Yenew, EPHI, (Addis Ababa, Arbegnoch Street, P.O. Box 1242)

PI of the project

Phone: +251921941156 or

Tel address: +25111-2780845

Email: bazezewyenew@yahoo.com

Endale Mengesha, KNCV Tuberculosis Foundation, (Addis Ababa፣ Bole subcity)

First co-investigator

Phone: +251910009500

Email: endalemengesha.goshu@kncvtbc.org

For any ethical complaint and your right

Ethiopian Public Health Institute/EPHI

Scientific and Ethic Review Office /SERO, Addis Ababa, Arbegnoch Street, P.O.Box 1242

EPHI-IRB Tel: +251 118685503/15

Do you agree for your child to participate in the project?

☐ Yes, I agree for my child to participate in the study

☐ No, I do not wish for my child to participate in the study

Participant's **parents/guardians'** statement:

The above survey has been explained to me and my child, and I agree that my child to take part in the survey. I understand that this is my choice and that if I change my mind, I can decide to end the interview at any time.

Participant's parents/guardians name and signature………………………. Date…………………

Interviewer name and signature….……………………………………….Date…………………
