# Supplementary material for: The Simple One-step stool processing method for detection of Pulmonary tuberculosis: A study protocol to assess the robustness, stool storage conditions and sampling strategy for global implementation and scale-up
Source: PLoS One. 2022 Oct 4;17(10):e0264103. doi: 10.1371/journal.pone.0264103 (PMC9531811; doi:10.1371/journal.pone.0264103)
Supplement: S2 Appendix — (PDF) [file pone.0264103.s002.pdf]

Appendix 2: PODTEC Stool Submission Form (copy stays at clinic, original submitted to EPHI)

EPHI: put PODTEC barcode here

Clinic: put ASTTIE UPIC barcode here

**GENERAL INFORMATION,** *TO BE FILLED BY CLINIC SITE COORDINATOR*		
**Name of clinic:**	____________________________	
Name of person preparing stool sample for submission:	____________________________	
Cell phone number:	____________________________	
Name of participant:	____________________________	
Participant eligible because Xpert-Ultra MTB-positive:	☐ on sputum/NGA<br>☐ on stool	*tick all options that apply*<br>*note: if no option applies, the child is not eligible for PODTEC*
When did the child start TB treatment?	_ _ /_ _/ _ _ _ _<br>DD / MM / YYYY<br>☐ treatment has not (yet) started	*note: if it is >5 days after start of TB treatment, the stool cannot be submitted*
What is the stool number submitted?	○ first stool (used for diagnosis in ASTTIE project)<br>○ second stool<br>○ third stool	*tick only one option*
**INFORMATION RETRIEVED FROM PARENT/CAREGIVER, TO BE FILLED BY NURSE RECEIVING SAMPLE**		
Date and time that stool was collected	_ _ /_ _/ _ _ _ _ DD / MM / YYYY _ _ : _ _ HH : MM	
Where was the stool collected?	○ on-the-spot in clinic, collected by nurse<br>○ on-the-spot in clinic, collected by caregiver<br>○ at the home of the caregiver	→ *go to next page*<br>→ *go to next page*
Where was the sample stored before it was brought to the clinic?	○ at room temperature: _ _ °C*<br>○ in the caregiver's refrigerator	*tick only one option*<br>**enter estimated temperature*

How long did the caregiver travel from his/her home to the clinic?	_ _ : _ _<br>HH : MM		*enter estimated time*
Where were the samples kept during the transport?	O at outside temperature:<br>O other, specify:<br>___________________________	_ _ ºC*<br>_ _ ºC*	*tick only one option*<br>**enter estimated temperature*
Date and time of **sample receipt from parent/caregiver**:	_ _ /_ _/ _ _ _ _<br>DD / MM / YYYY	_ _ : _ _<br>HH : MM	
**INFORMATION RETRIEVED FROM LABORATORY STORING STOOL BEFORE TRANSPORTATION**			
Before **sample pick-up by EPSE**, the sample was stored:	O at room temperature:<br>O in the refrigerator	_ _ ºC*	*tick only one option*<br>**enter estimated temperature*
Date and time of sample **pick-up by EPSE**:	_ _ /_ _/ _ _ _ _<br>DD / MM / YYYY	_ _ : _ _<br>HH : MM	
**INFORMATION TO BE FILLED BY EPHI LABORATORY TECHNICIAN**			
Date and time of sample **received at EPHI**:	_ _ /_ _/ _ _ _ _<br>DD / MM / YYYY	_ _ : _ _<br>HH : MM	
Was the sample transported in cold-chain?	O yes, from end-to-end<br>O yes, partly<br>specify: ________________________<br>O no<br>O unknown	 _ _ ºC*<br><br>_ _ ºC*	*tick only one option*<br>**enter estimated temperature*<br>*note: option "unknown" should be avoided by asking EPSE courier for transport conditions*
What is the stool number **received according to EPHI information?**	O remainder of first stool *(this is the sample used for the diagnosis of TB)*<br>O second sample<br>O third sample		*tick only one option*
Appearance of the stool sample:	O formed (solid)<br>O unformed (soft)<br>O taking the shape of the container (liquid)		*tick only one option*
Stool weight	___ gram		*please measure!*
