# Supplementary figures and images for: The Simple One-step stool processing method for detection of Pulmonary tuberculosis: A study protocol to assess the robustness, stool storage conditions and sampling strategy for global implementation and scale-up
Source: PLoS One. 2022 Oct 4;17(10):e0264103. doi: 10.1371/journal.pone.0264103 (PMC9531811; doi:10.1371/journal.pone.0264103)

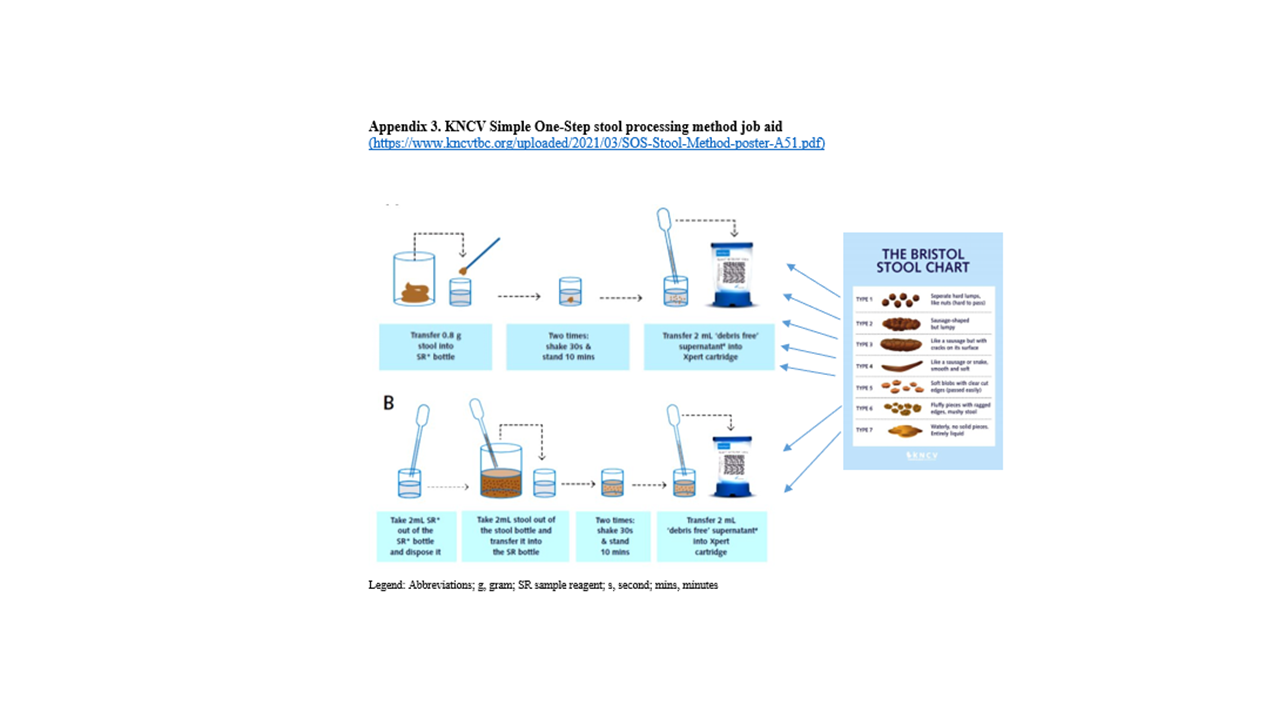

Supplement: S3 Appendix — (TIF) [file pone.0264103.s003.tif]
