# Supplementary material for: The Simple One-step stool processing method for detection of Pulmonary tuberculosis: A study protocol to assess the robustness, stool storage conditions and sampling strategy for global implementation and scale-up
Source: PLoS One. 2022 Oct 4;17(10):e0264103. doi: 10.1371/journal.pone.0264103 (PMC9531811; doi:10.1371/journal.pone.0264103)
Supplement: S4 Appendix — (PDF) [file pone.0264103.s004.pdf]

Appendix 4: Laboratory experiment result capture form*

EPHI: put PODTEC barcode here

1. Stool sample number	O 1 (first sample)<br>O 2<br>O 3	*see middle part of code on barcode sticker on aliquot pot*
2. Aliquot number	_ _	*see last part of code on barcode sticker on aliquot pot*
3. Initials of laboratory technician conducting test:	_____	
4. Remarks on stool appearance:	O looks normal<br>O overgrown with fungi<br>O gas formation has caused lid to drop off<br>O other, specify:<br>____	*tick only one*
5. Experiment number:	O 1a (sample strategy: 3 different stools) → *Q10*<br>O 1b (sample strategy: 3 aliquots of 1 stool) → *Q10*<br>O 2 (storage conditions) → *Q6*<br>O 3a (processing conditions) → *Q7*<br>O 3b (maximum mixture storage time) → *Q8*<br>O 3c (optimum amount of stool processed) → *Q9*	*tick only one*
6. a. Sample storage temperature	O Room (20-22^{0}C)<br>O Incubator (35-37^{0}C)<br>O Refrigerator (2-6^{0}C)<br>O Freezer (-20^{0}C)	*tick only one option*
b. Sample storage time	O 4-6 hours<br>O 24 hours<br>O 72 hours (3 days)<br>O 120 hours (5 days)	*tick only one option*<br><br>*After filling this question:* → ***Q10***

7. a. Sample + SR buffer mixture processing time	○ 10’ + 10’<br>○ 10’ + 5’<br>○ 15’<br>○ 10’ + 10’ vortex		*tick only one option*
8. a. What was the contact time between sample and SR after initial mixing?	○ 2 hours<br>○ 5 hours<br>○ 24 hours		*tick only one option*
b. At what temperature was the sample/SR mixture stored?	○ Room (20-22^{0}C)<br>○ Refrigerator (2-6^{0}C)		*tick only one option*<br>*After filling this question:* → ***Q10***
9. Amount of stool tested	○ 0.3 g<br>○ 0.5 g<br>○ 0.8 g<br>○ 1.0 g<br>○ 1.2 g		*tick only one option*
10. Date and time of stool processing for Xpert-Ultra started	_ _ /_ _/ _ _ _ _<br>DD / MM / YYYY	_ _ : _ _<br>HH : MM	
11. Date and time of Xpert-Ultra started	_ _ /_ _/ _ _ _ _<br>DD / MM / YYYY<br><br>☐ test not started, reason:<br>________________________	_ _ : _ _<br>HH : MM	
12. Xpert-Ultra result	○ MTB trace detected<br>○ MTB detected very low<br>○ MTB detected low<br>○ MTB detected medium<br>○ MTB detected high<br>○ MTB not detected<br>○ Error code: _ _ _<br>○ Invalid<br>○ No result	○ RIF resistant<br>○ RIF susceptible<br>○ RIF indeterminate	*tick only one option for both the MTB and the RIF result*

13. Cycle threshold (Ct) values	Probe A: _ _	Probe E: _ _
	Probe B: _ _	Probe IS6110/IS1081: _ _
	Probe C: _ _	SPC: _ _
	Probe D: _ _	
