# Supplementary material for: The Simple One-step stool processing method for detection of Pulmonary tuberculosis: A study protocol to assess the robustness, stool storage conditions and sampling strategy for global implementation and scale-up
Source: PLoS One. 2022 Oct 4;17(10):e0264103. doi: 10.1371/journal.pone.0264103 (PMC9531811; doi:10.1371/journal.pone.0264103)
Supplement: Attachment — Submitted filename: Feedback to the reviewers_final_24Aug2022.docx [file pone.0264103.s006.docx]

Feedback to the reviewer

We would like to thank the reviewers for their positive response on the revised version of the manuscript. It was nice to hear that the protocol has greatly improved. We herewith like to provide feedback to the last comment provided by reviewer 1

“The above manuscript is greatly improved int the revised version. I am mostly satisfied with your response to the reviewer’s concerns. The protocol carries much better clarity. However, right statistics do play a major role in determining the sample size and the analysis of the proposed study. I am not sure if the use of Statulator.com is enough for such studies. I do like to see the study established in consultation with an expert statistician, especially if enrolling a small number of MTB confirmed negative patients might help them establish their specificity. I understand the financial and resource limitations, but strongly recommend it, as this would be very important in understanding the application of the proposed method for routine use. Therefore, I am recommending minor revision to the protocol.”

*Reply: Though not explicitly stated on their website, Statulator seems to make use of Mcnemar’s two-sample paired proportions test to estimate sample sizes. We consulted Dr L. Stuck at the university of Amsterdam, a statistician, epidemiologist and data scientist (*[*https://www.aighd.org/people/logan-stuck/*](https://eur01.safelinks.protection.outlook.com/?url=https%3A%2F%2Fwww.aighd.org%2Fpeople%2Flogan-stuck%2F&data=05%7C01%7C%7C6d332f056ef049e8572008da85ab8d24%7Cf28ee6e5b8a648c589d0aaeef9ebd92e%7C0%7C0%7C637969269096105833%7CUnknown%7CTWFpbGZsb3d8eyJWIjoiMC4wLjAwMDAiLCJQIjoiV2luMzIiLCJBTiI6Ik1haWwiLCJXVCI6Mn0%3D%7C3000%7C%7C%7C&sdata=bdfYfYRUIKJ8foFO2WqCUS%2FhdDNQE7RwSekfmx1DGWQ%3D&reserved=0)*), who confirmed that this is the correct test to use for sample size calculations. For reproducibility, we repeated the sample size calculations in Stata using McNemar’s test. This can be done using the “power paired proportions” command. We have adapted the title of Figure 4 accordingly.*
